# A novel mutation in RAB3GAP1 gene in Chinese patient causing the Warburg micro syndrome: A case report

**DOI:** 10.1097/MD.0000000000022902

**Published:** 2021-01-15

**Authors:** Dan Zhou, Qiu Wang, Hanmin Liu

**Affiliations:** a Department of Pediatrics, West China Second University Hospital, Sichuan University; b Key Laboratory of Birth Defects and Related Diseases in Women and Children, Sichuan University, Ministry of Education; c Department of Rehabilitation Medicine, West China Second University Hospital, Sichuan University, Chengdu, Sichuan, China.

**Keywords:** congenital cataracts, developmental delay, gene mutation, RAB3GAP1, Warburg micro syndrome

## Abstract

**Rationale:**

Warburg Micro syndrome is a rare, autosomal recessive disorder characterized by multiple organ abnormalities involving the ocular, nervous, and genital systems. This case report describes a novel mutation in the *RAB3GAP1* gene associated with Warburg Micro syndrome.

**Patient concerns:**

A 6-month-old female infant with bilateral congenital cataracts and developmental delay was referred to our department for further assessment. She presented with facial dysmorphic features, including a prominent forehead, microphthalmia, wide nasal bridge, relatively narrow mouth, large anteverted ears, and micrognathia.

**Diagnoses:**

The patient was diagnosed with Warburg Micro syndrome based on clinical manifestations, as well as a novel homozygous mutation in *RAB3GAP1:* c.75–2A>C. Both parents were identified as heterozygotic carriers of this mutation.

**Interventions:**

Bilateral cataract extraction and anterior vitrectomy were performed at age 6 months, followed by physical rehabilitation. Convex lenses were used to protect the eyes postoperatively until intraocular lens implantation.

**Outcomes:**

Although the patient received physical rehabilitation, she suffered global developmental delay.

**Lessons:**

The c.75–2A>C mutation in *RAB3GAP1* expands the spectrum of known mutations in this gene, and it may be associated with Warburg Micro syndrome. Genetic counselors may wish to take this finding into consideration, especially given the poor prognosis associated with the disease.

## 1 Introduction

Warburg Micro syndrome (OMIM 600118) is a rare, autosomal recessive genetic disorder^[1]^ characterized by congenital cataracts, microphthalmia, postnatal microcephaly, corpus callosum hypoplasia, microcornea, progressive joint contractures, hypothalamic hypogonadism, and severe developmental delay with growth failure.^[2–4]^ The disease has been associated with mutations in the Ras-associated binding 3 GTPase-activating protein 1 (RAB3GAP1), encoded by a gene at chromosome 2q21.3 (MIM ∗602536).^[5]^ In fact, 70 nonsense, missense, frameshift, and splice site mutations in *RAB3GAP1* have been associated with Warburg Micro syndrome (Clinvar, https://www.ncbi.nlm.nih.gov/clinvar/).

Here we report a Chinese patient with Warburg Micro syndrome associated with an *RAB3GAP1* mutation never described before.

## 2 Ethics approval

Written informed consent was obtained from the parents of the patient for her anonymized clinical and genetic data to be analyzed and published for research purposes. This study was approved by the Ethics Committee of the West China Second Hospital of Sichuan University, Chengdu, China.

## 3 Case report

### 3.1 Patient characteristics

**Figure 1:**
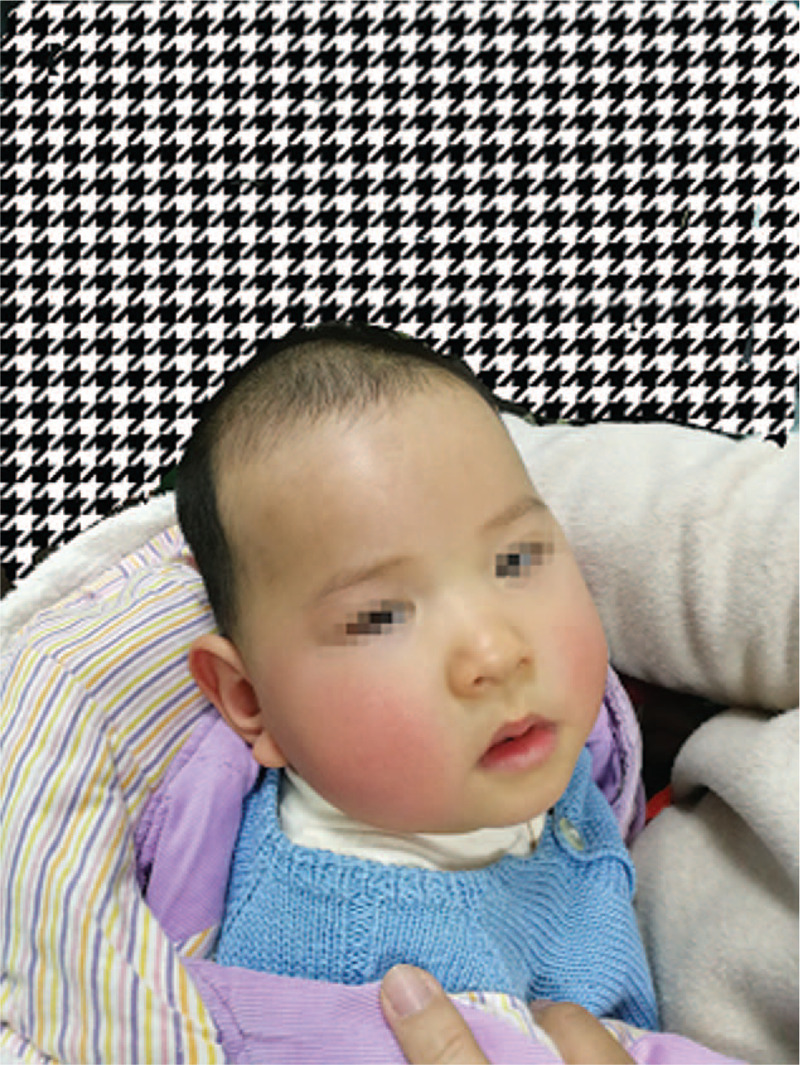
A 6-month-old Chinese girl presenting with typical dysmorphic facial features, including a prominent forehead, wide nasal bridge, relatively narrow mouth, large anteverted ears, and micrognathia.

A 6-month-old infant girl was referred to our hospital with bilateral congenital cataracts and severe developmental delay. At admission, the patient was unable to support her head or maintain a sitting position without assistance. Further physical examination showed a range of dysmorphic facial features, including a prominent forehead, wide nasal bridge, relatively narrow mouth, large anteverted ears, and micrognathia (Fig. 1).

The patient had been born at full term (39 weeks) after a normal pregnancy. At birth, the infant weighed 2900 g (25–50th percentile), was 50 cm long (50–75th percentile) and had an occipitofrontal head circumference of 33 cm (25–50th percentile). Soon after birth, ocular abnormalities were observed, such as microphthalmia, unresponsive pupils, microcornea, and bilateral cataracts, resulting in complete blindness. Additionally, she had poor axial hypotony, poor straightening, poor vertical control of the head, as well as lower limb spasticity, but without seizures.

The child had been born to a 37-year-old mother and non-consanguineous 51-year-old father, each of whom had already had one healthy son with a previous partner. The neither parent reported a family history of genetic diseases.

### 3.2 Medical examination

#### 3.2.1 Laboratory investigation

Normal results were obtained for thyroid function, liver function, renal function, vitamin D, serum calcium, creatine kinase, creatine kinase isoenzyme, ammonia, lactate, beta hydroxybutyric acid, pyruvate, and routine blood tests. Urine tested negative for mucopolysaccharide storage. Routine chromosomal analysis showed a normal female karyotype.

#### 3.2.2 Brain imaging

**Figure 2:**
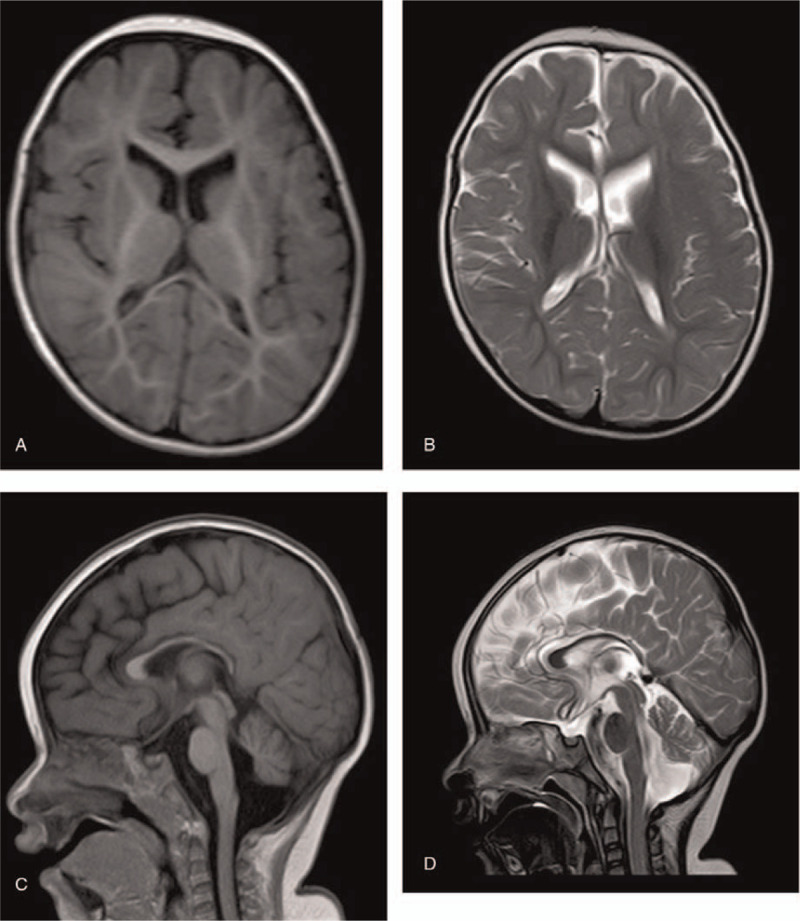
Magnetic resonance imaging of the patient's brain at 17 months A-D, which shows a sylvian fissure that is wider and deeper than normal, delayed myelinization, mild hypoplasia of the corpus callosum, and cerebellar hypoplasia.

**Figure 3:**
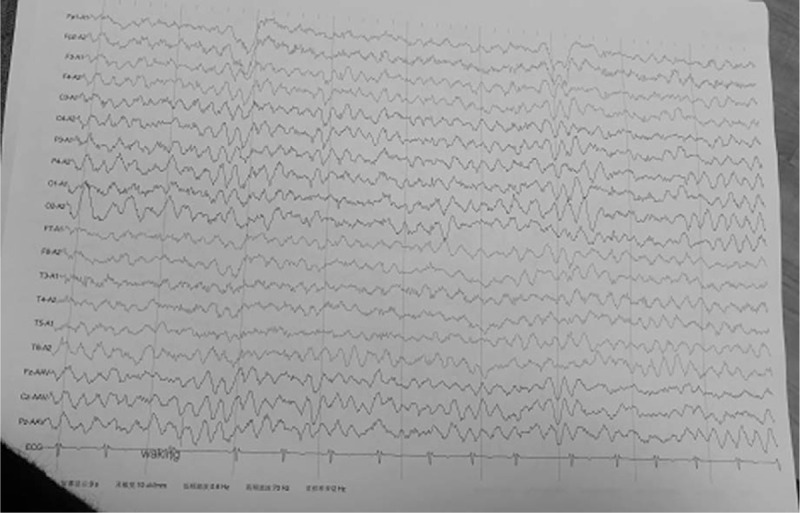
Electroencephalogram at 11 months, showing normal brain activity.

Magnetic resonance imaging showed cortical malformations (Fig. 2A–D), including a wider and deeper sylvian fissure, delayed myelinization, mild hypoplasia of the corpus callosum, and cerebellar hypoplasia. The electroencephalogram showed normal brain activity (Fig. 3).

#### 3.2.3 Genetic testing

**Figure 4:**
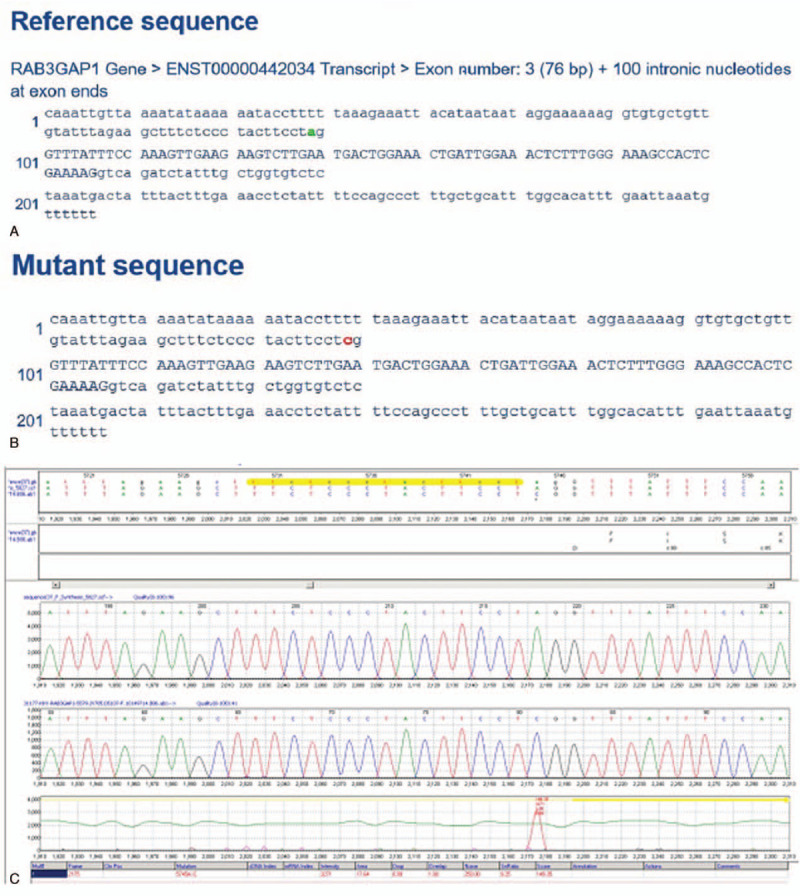
Family pedigrees and mutations: A, depicts the reference sequence, B, the mutant sequence, and C, the novel homozygous mutation c.75–2A>C in intron 2 of *RAB3GAP1*.

Analysis of patient revealed one homozygous change in *RAB3GAP1* relative to the reference sequence NM_001172435: the splice site mutation c.75–2A>C in intron 2 (Fig. 4A–C). Both parents were found to be heterozygous carriers of the mutation. Copy number and single-nucleotide polymorphism analyses suggested that the patient did not have a variation in copy number. Sequencing of the complete mitochondrial genome of the patient and her mother did not identify any changes potentially associated with the patient's condition.

Various software packages predicted that the c.75–2A>C mutation affects splicing, which could lead to a truncated, catalytically inactive protein. We classify this mutation as a Class 2 variant that is likely pathogenic, based on the guidelines of the American College of Medical Genetics (PMID: 25741868). This mutation appears not to have been reported yet, and it is not listed in the Human Gene Mutation Database (HGMD).

### 3.3 Diagnosis

The patient was diagnosed with Warburg Micro syndrome based on clinical manifestations, additional medical examinations, and the novel homozygous c.75–2A>C mutation in *RAB3GAP1*.

### 3.4 Treatment

Bilateral cataract extraction and anterior vitrectomy on the infant at 6 months, followed by physical rehabilitation. After surgery, her eyes were protected with convex lens, then intraocular lenses were implanted.

### 3.5 Follow-up

Although the patient received physical rehabilitation, she suffered global developmental delay. During follow-up, at age 11 months, the patient weighed 8200 g (10–25th percentile), was 72 cm tall (10–25th percentile), and had an occipitofrontal head circumference of 41.8 cm (3–10th percentile). She showed mild hearing loss and postnatal microcephaly, and was unable to support her head or sit without assistance. Although the physical rehabilitation helped eliminate muscle rigidity, she continued to show high muscle tone in her lower limbs.

Assessment using the Bayley Scales of Infant Development-2 showed a psychomotor development index of 23 (<0.1% of her peers), equivalent to the level of a 3-month-old infant. Her mental development index was 22 (<0.1% of her peers), equivalent to the level of a 2-month-old infant.

## 4 Discussion

Warburg Micro syndrome, also called Micro syndrome, was first described in 1993.^[1]^ So far, mutations in four genes that encode Ras-associated binding proteins and regulators have been associated with this syndrome: *RAB3GAP1* (NM_001172435),^[5,6]^*RAB3GAP2* (NM_012414),^[7]^*RAB18* (NM_001256410)^[8]^ and *TBC1D20* (NM_144628).^[9]^ The syndrome appears to occur most frequently in association with *RAB3GAP1* mutations, which occur in approximately 40% of all patients.^[10]^

Seven transcripts have been identified from the *RAB3GAP1* gene. The major transcript (ENST00000264158) contains 24 exons. The major transcript in our patient (ENST00000442034), however, contained an additional exon encoding the RAB3 GTPase-activating protein. This protein aids in the conversion of RAB3 from its active GTP-bound form to its inactive GDP-bound form.^[5]^ RAB3 proteins help regulate exocytosis of neurotransmitters and hormones that are essential for normal eye and brain development. ^[11,12]^

The c.75–2A>C in *RAB3GAP1* intron 2 of our patient is located in a highly conserved splice site, and it is predicted to affect normal mRNA splicing and lead to a truncated, catalytically inactive protein. The present report extends the range of *RAB3GAP1* mutations that generate truncated forms of the protein lacking some or all of the catalytic domain.^[3,5,6,10]^ The fact that these various mutations have the same effect of inactivating the protein may explain why they all lead to a similar clinical phenotype. This relatively low phenotypic variation observed across patients with different mutations in RAB3GAP1 can be explained by the formation of truncated proteins, which occur before or within the region important for catalytic activity.

Our patient showed many typical features associated with this syndrome, including congenital cataracts, microphthalmia, postnatal microcephaly, corpus callosum hypoplasia, microcornea, limb spasticity, and severe developmental delay with growth failure. However, magnetic resonance imaging did not show the cortical dysplasia, such as frontal and parietal polymicrogyria, typical of other patients with Warburg Micro syndrome. In addition, our patient showed milder hypoplasia in the corpus callosum than other patients with the disease. Further studies are essential to clarify and explain differences in clinical manifestations among patients with different *RAB3GAP1* mutations.

Genetic analyses may be particularly useful for differential diagnosis of Warburg Micro syndrome and Martsolf syndrome (OMIM 212720), particularly in patients with unknown medical history. Both syndromes are autosomal recessive and involve congenital cataracts, microphthalmia, postnatal microcephaly, and developmental delay. However, Martsolf syndrome appears to be even rarer and to involve less severe neurodevelopmental and ophthalmological defects.^[1,13]^

## 5 Conclusion

We describe a novel homozygous splice site mutation in *RAB3GAP1* associated with Warburg Micro syndrome in a Chinese patient. The identification of such mutations is essential for accurate genetic counseling. Further in vivo and in vitro studies should provide a better understanding of the functional and clinical effects of *RAB3GAP1* mutations.

## Acknowledgments

The authors thank the patient and her family for providing informed consent for the publication of this case report.

## Author contributions

**Conceptualization:** Han Min Liu

**Investigation:** Dan Zhou

**Resources:** Dan Zhou

**Supervision:** Han Min Liu

**Writing – original draft:** Dan Zhou, Qiu Wang

**Writing – review & editing:** Dan Zhou, Qiu Wang

## Abbreviations

Abbreviation: RAB3GAP1 = Ras-associated binding 3 GTPase-activating protein 1.
